# Primary Rhabdomyosarcoma of Kidney with Local Recurrence and Liver Metastasis in Adults: A Case Report

**DOI:** 10.15586/jkcvhl.v9i1.218

**Published:** 2022-04-14

**Authors:** Hamid Nasrollahi, Ali Eslahi, Ali Ariafar, Faisal Ahmed, Ahmad Monabati

**Affiliations:** 1 Radiation Oncology Department, School of Medicine, Shiraz University of Medical Sciences, Shiraz, Iran;; 2 Department of Urology, School of Medicine, Shiraz University of Medical Sciences, Shiraz, Iran;; 3 Urology Research Center, Al-Thora General Hospital, Department of Urology, School of Medicine, Ibb University of Medical Sciences, Ibb, Yemen;; 4 Department of Pathology, School of Medicine, Shiraz University of Medical Sciences, Shiraz, Iran

**Keywords:** adult, case report, kidney, liver metastasis, pleomorphic, rhabdomyosarcoma, disease progression

## Abstract

Primary rhabdomyosarcoma (RMS) of the kidney in an adult is rare, with only a few cases published in the literature. It is a mesenchymal tumor associated with an aggressive and rapid clinical progression course. We present a case of primary renal RMS in a 58-year-old female who presented with intermittent abdominal pain in the past year. The computed tomography (CT) scan revealed a 20×25×8 cm heterogeneous solid mass in the middle pole extended to the lower pole of the right kidney. Therefore, the patient underwent a right radical nephroureterectomy. Histopathology examination and immunohistochemistry studies confirmed the diagnosis of RMS with pleomorphic components. Postoperatively, the patient was discharged without any complications and was referred to an oncologist for chemotherapy. However, a follow-up CT scan in 2 months showed widespread liver metastasis and local recurrence. The patient received Gemcitabine and Docetaxel, but her condition worsened, and she passed away 5 months later. Primary renal RMS is rare in adults. In addition, liver metastasis is uncommon and poorly understood. Hence, we describe the clinicopathologic characteristics, including clinical follow-up of our case, focusing on the disease progression, treatment, and outcome.

## Introduction

Rhabdomyosarcoma (RMS) of the kidney is a distinctive form of renal sarcoma that develops from the skeletal muscle progenitor cells. It occurs only in 1–3% of primary renal malignancies (1). Embryonal, alveolar, and pleomorphic variations are among the histological subtypes. Primary pleomorphic RMS in adults are exceedingly uncommon (2, 3). In addition, in adulthood, these tumors appear late and aggressively, with the majority of cases developing metastasis at the time of diagnosis (2). Hence, we describe our patient’s clinicopathologic characteristics, including clinical follow-up, focusing on the disease treatment and outcome.

## Case Report

A 58-year-old female visited our urology clinic due to intermittent abdomen pain in the past year. Her pain was not colicky and did not interfere with her routine life. She had no other urologic problem, such as dysuria, frequency, or incontinence. Her medical history was not significant, with no history of smoking.

**Figure 1:**
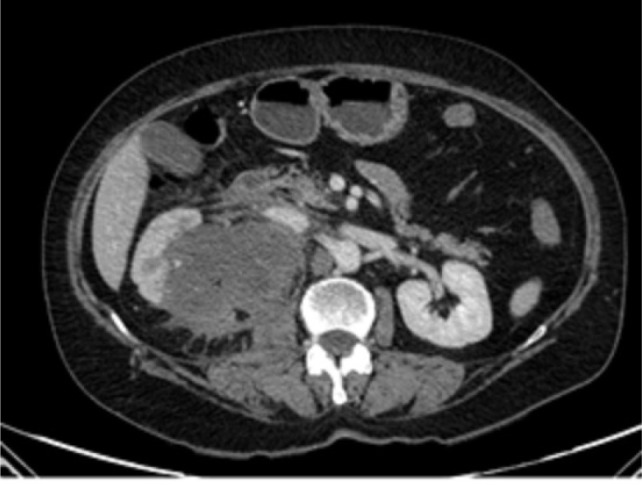
CT scan of the abdomen demonstrates the right renal mass.

Physical examination revealed only mild right flank tenderness with right palpable, nonmobile mass. Urine analysis showed microscopic hematuria (15/20 RBCs/HPF). The routine blood tests were within the normal range. Ultrasonography (US) showed a 20×25 cm solid hypoechoic mass in the right med and lower pole of the kidney. A chest, abdomen, and pelvic computed tomography (CT) scan was done, which showed a 20×25×8 cm heterogeneous solid mass in the middle pole that extended to the lower pole of the right kidney with delayed enhancement . There was no evident local invasion or metastases in the chest CT scan. However, the tumor was pressing the liver, and no defined fat planes were seen (Figure 1).

The patient underwent a right radical nephroureterectomy. Histopathology examination studies confirmed the diagnosis of RMS with a pleomorphic component that was limited to the kidney. The ureter, perinephric fat, adrenal gland, and vascular margin were free of tumor. There were no lymphovascular invasions. However, tumor necrosis was presented. Light microscopy disclosed a malignant tumor made up of large, haphazardly arranged cells of various shapes with abundant, intensely eosinophilic cytoplasm. Cytoplasmic cross-striations were discovered in the spindle and tadpole-shaped cells. There were different mitotic figures and necrotic areas (Figur 2). Immunohistochemistry revealed that myogenin, smooth muscle actin (SMA), and desmin stained positively, but cytokeratin, EMA, CD10, S100 protein, and vimentin stained negatively. These findings indicated that the tumor arose from the skeletal muscle, leading to the right kidney and the final diagnosis of pleomorphic RMS (Figure 3).

**Figure 2:**
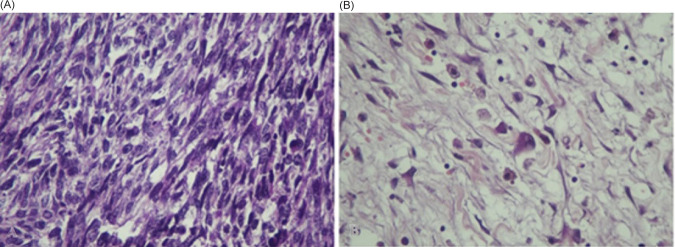
(A) H&E section from tumor show malignant spindle cells with hyperchromatic nuclei. (B) H&E section show some atypical spindle and round cells with abundant eosinophilic cytoplasm (Rhabdomyolysis differentiation).

**Figure 3:**
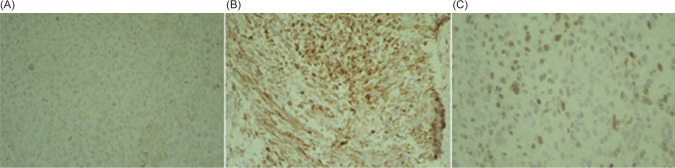
Immunohistochemistry positive reaction for (A) Creatine kinase. (B) Desmin. (C) Myogenin.

**Figure 4:**
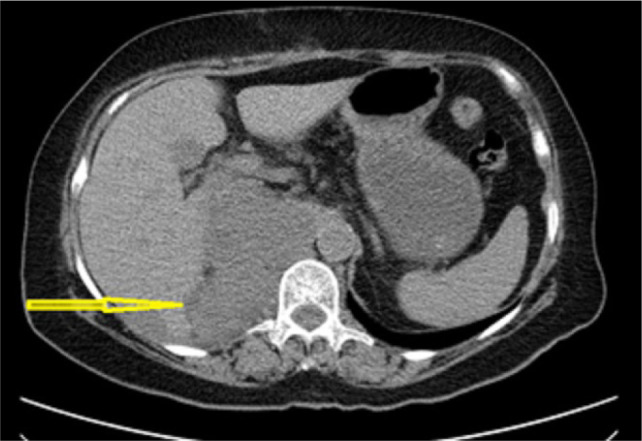
Postoperative abdominopelvic CT scan demonstrates local recurrence and liver metastasis (Arrow).

Postoperatively, the patient showed unremarkable recovery. Two months after surgery, the patient received chemotherapy (Adriamycin, Ifosfamide, and Vincristine) and radiotherapy, but an abdominopelvic CT scan showed widespread liver metastasis and local recurrence (Figure 4). The patient’s general condition worsened, and she passed away 5 months later.

## Discussion

RMS is the least common type of cancer mentioned in the literature among all adult sarcomas. It accounts for between 2 and 5% of all adult soft tissue cancers, with fewer than 20% occurring in the urogenital organs. It was initially classified into four histological categories: embryonal, botryoid-subtype of embryonal, alveolar, and pleomorphic (4). However, it is apparent that pleomorphic RMS acts aggressively and appears late, as do other primary sarcomas. The average age at diagnosis for kidney sarcomas is 49 years, and the average size is between 55 and 230 mm (5). Our case involved a 58-year-old female, and the mass size was 20 cm.

Primary renal RMS is hard to diagnose. According to Grignon et al., the requirements for diagnosing renal sarcomas comprise three main components. To begin with, there must be no evidence of sarcoma in another place to rule out metastatic tumors. Second, a sarcomatoid renal cell carcinoma (RCC) must be ruled out by taking sufficient tumor samples to rule out an epithelial component. Finally, histology can rule out the spread of a retroperitoneal sarcoma with subsequent renal invasion (6).

The positron emission tomography (PET) scan is superior to conventional CT scan to rule out the potential competing primary source of RMS cases (7). However, in our patient, the perioperative CT scan showed the renal mass without any evidence of metastasis. The PET scan was not performed due to the high cost of this modality and the low economic status of the patient. A recent study by Harrison et al. reported that PET scan does not predict event-free survival in intermediate-risk or high-risk RMS patients (8).

In most instances of RMS, the expressions of myogenic regulatory factors, such as Desmin and MyoD1, and vimentin are positive. MyoD1 is a muscle regulatory transcription factor located in an RMS marker’s nucleus. However, a subset of pleomorphic RMS patients shows exclusively cytoplasmic MyoD1 expression due to myogenic transactivation. The cross-reactivity with unidentified proteins in the cytoplasm makes it less selective for RMS. It can also be detected in renal angiomyolipomas, perivascular epithelioid cell tumors, neuroblastomas, and peripheral primitive neuroectodermal tumors. Other stains are acquired to distinguish RMS from different renal cancers (9).

The initial tumor location is managed with surgical excision and radiotherapy, while chemotherapy manages metastasis. Chemotherapy is a critical therapeutic strategy for preventing tumor spread (10). Chemotherapeutic agents include Leurocristine, Dactinomycin, Cyclophosphamide Doxorubicin, Mitoxantrone, Toposar, and Hycamtin. Factors associated with poor prognosis are the presence of metastasis at presentation and weak response to neoadjuvant chemotherapy agents (11). Our patient was given Adriamycin, Ifosfamide, and Vincristine, but she did not respond well. Her general condition worsened, and she passed away after 5 months. The effect of vascular invasion on the survival rate of RMS cases is still unclear. Nevertheless, Lin et al. presented a case of adult renal RMS with rapid lung metastases due to vascular invasion and incomplete resection of the primary tumor (9).

Adult kidney RMS survival rate information is scarce due to its rarity. The adult kidney RMS 5-year overall survival rate is 17.1% (range: 2.9–41.6%), with a higher incidence of mortality (12). Fang et al. described a primary right renal RMS in an adult woman who suffered from hematuria and colic pain. An abdominal CT scan showed a 5.4×4.3 cm mass located in the upper pole of the right kidney without evidence of metastasis. However, renal capsular invasion and invasion of the ureter were discovered during surgery. After the initial postoperative diagnosis, chemotherapy with Vincristine, Actinomycin D, Cyclophosphamide, and radiotherapy was started. However, no longer follow-up was provided (only 4 months) (13).

Furlong et al. reported 38 cases of pleomorphic RMS in adults. In a follow-up of 30 (79%) cases, it was discovered that 70% of cases expired with a survival rate of 20 months (range: 1–108 months); 3% of cases were still alive at 12 months, and 27% were free of disease. Within a mean of 9 months (range: 2–24 months), 45% of the cases developed with their first local recurrence, with up to two recurrences reported. Within a 15-month average follow-up, 45% of cases developed metastases (range: 36 months) (14). Our patient developed liver metastasis and local recurrence within 2 months and died 5 months after surgery.

## Conclusion

Primary pleomorphic RMS of the kidney is rare and vastly aggressive, with a low survival rate in the adult population. It seems that the RMS, even after successful total resection and adjuvant therapies, still has a high rate of local recurrence, metastasis, and mortality.

## Consent

Written informed consent was obtained as per institutional guidelines.
